# Integrated analysis of lncRNA–miRNA–mRNA ceRNA network and the potential prognosis indicators in sarcomas

**DOI:** 10.1186/s12920-021-00918-x

**Published:** 2021-03-02

**Authors:** Lu Gao, Yu Zhao, Xuelei Ma, Ling Zhang

**Affiliations:** 1 College of Medicine, Southwest Jiaotong University, Chengdu, 610031 Sichuan China; 2 Department of Oncology, The General Hospital of Western Theater Command, Chengdu, 610083 Sichuan China; 3 Department of Biotherapy, West China Hospital and State Key Laboratory of Biotherapy, Sichuan University, Chengdu, 610041 Sichuan China

**Keywords:** Sarcomas, CeRNA network, Overall survival, Prognosis indicators

## Abstract

**Background:**

Competitive endogenous RNA (ceRNA) networks have revealed a new mechanism of interaction between RNAs, and play crucial roles in multiple biological processes and development of neoplasms. They might serve as diagnostic and prognosis markers as well as therapeutic targets.

**Methods:**

In this work, we identified differentially expressed mRNAs (DEGs), lncRNAs (DELs) and miRNAs (DEMs) in sarcomas by comparing the gene expression profiles between sarcoma and normal muscle samples in Gene Expression Omnibus (GEO) datasets. Gene ontology (GO) and Kyoto encyclopedia of genes and genomes (KEGG) pathway enrichment analyses were applied to investigate the primary functions of the overlapped DEGs. Then, lncRNA-miRNA and miRNA-mRNA interactions were predicted, and the ceRNA regulatory network was constructed using Cytoscape software. In addition, the protein–protein interaction (PPI) network and survival analysis were performed.

**Results:**

A total of 1296 DEGs were identified in sarcoma samples by combining the GO and KEGG enrichment analyses, 338 DELs were discovered after the probes were reannotated, and 36 DEMs were ascertained through intersecting two different expression miRNAs sets. Further, through target gene prediction, a lncRNA–miRNA–mRNA ceRNA network that contained 113 mRNAs, 69 lncRNAs and 29 miRNAs was constructed. The PPI network identified the six most significant hub proteins. Survival analysis revealed that seven mRNAs, four miRNAs and one lncRNA were associated with overall survival of sarcoma patients.

**Conclusions:**

Overall, we constructed a ceRNA network in sarcomas, which might provide insights for further research on the molecular mechanism and potential prognosis biomarkers.

## Background

Sarcomas are rare types of malignant neoplasms of mesenchymal origin and often spread to other tissues in the body [1]. Sarcomas can be classified into more than 100 distinct subtypes, which are typically divided into two heterogeneous groups, including bone sarcomas and soft tissue sarcomas [2, 3]. Despite the use of extensive surgical resection, intensive and multiagent chemotherapy, radiotherapy, and emerging treatment strategies consisting of molecular targeting agents, immune checkpoint inhibitors, and adoptive T-cell therapy, the overall 5-year survival rate of sarcomas has not been significantly improved over the past few decades [2, 4, 5]. Unfortunately, sarcoma patients accompanied with metastatic spread show a lower 5-year overall survival rate [6]. Further investigations into the pathogenesis of sarcomas and identification of the novel prognostic biomarkers that facilitate the improvement of therapy and prognosis of sarcomas are urgently needed.

Noncoding RNAs (ncRNAs), such as long noncoding RNAs (lncRNAs) and microRNAs (miRNAs), function as key regulators of gene expression, their involvement in various human diseases is being gradually revealed, and the multilayered regulatory networks formed by cross-linked ncRNAs and mRNAs seemly provide new insights into their regulatory mechanism with regards to both physiology and pathology [7, 8]. The competing endogenous RNAs (ceRNAs) have been suggested to be involved in essential biological processes and play crucial roles in the initiation and development of neoplasms, and they potentially serve as diagnostic and prognosis markers or therapeutic targets [9]. miRNAs are small RNAs of 19–25 nucleotides in length that can guide the posttranscriptional repression of protein-coding genes by binding to their mRNAs [10]. The lncRNAs that harbor a miRNA response element can compete with other RNA transcripts and thus theoretically function as ceRNAs [11]. A single miRNA can regulate multiple target RNAs that contain the specific miRNA response element and these RNAs can be regulated by multiple miRNAs lay the foundation for the construction of a ceRNA network [12]. Recent studies have shown novel roles of ceRNA network in lung cancer, breast cancer, gastric cancer, esophageal adenocarcinoma, esophageal squamous cell carcinoma, cholangiocarcinoma, ovarian cancer, and uterine corpus endometrial carcinoma [13–21]. In addition, a previous study has constructed a ceRNA network and predicted the prognosis of soft tissue sarcoma recurrence [22]. However, the ceRNA network based on sarcoma and normal tissue samples has not been reported.

In the present study, the genes expression profiles of sarcomas in the Gene Expression Omnibus (GEO) database were integrated, and the differentially expressed genes (DEGs), lncRNAs (DELs) and miRNAs (DEMs) were identified. Gene ontology (GO) and Kyoto encyclopedia of genes and genomes (KEGG) pathway enrichment analyses were performed to screen out significant functional groups of DEGs. Thereafter, a ceRNA network consisting of lncRNA–miRNA–mRNA was constructed based on miRNA-mRNA and lncRNA-miRNA interactions. In addition, protein–protein interaction (PPI) network of the DEGs involved in the ceRNA network and prognostic analysis based on survival information of sarcoma patients were conducted to explore the effects of these transcripts on sarcomas.

## Methods

### Acquisition of available datasets and lncRNA annotation

Microarray data including high-throughput genes or miRNA expression profiling of sarcoma patients and normal samples were acquired from the GEO database (www.ncbi.nlm.nih.gov/geo/). The Affymetrix Human Genome U133 Plus 2.0 Array was reannotated according to the latest release of the updated gene database to filter lncRNA probes [23]. The process of lncRNA annotation was as follows: transcription sequences beginning with NM and XM in the RefSeq database were selected as a mRNA database, and ncRNAs with a length greater than 200 nucleotides that had gene transcription sequences beginning with NR and XR were selected from RefSeq, Ensembl and NONCODE2016 as a lncRNA database. Sequence alignments between 54,675 probes that were downloaded from the annotation file of the Affymetrix Human Genome U133 Plus 2.0 Array and the mRNA database were performed by using BLAST, and the E value was set as 10^–5^. Probes that match the respective gene were included in the mRNA probe set, and others were compared with the lncRNA database. The E value was set as 10^–5^, and probes that match the respective gene were included in the lncRNA probe set, which was use for the following analysis.

### Identification of DEGs, DELs and DEMs

DEGs, DELs and DEMs between sarcoma and normal muscle samples were determined by using the limma package in the R software (version 3.3.2) [24, 25]. *P* < 0.05, the false discovery rate (FDR) < 0.05 and fold change (FC) > 3 was used as the screening criteria for DEGs and DELs, while *P* < 0.05, FDR < 0.05 and FC > 2 was used to screen DEMs. Hierarchical clustering was performed using EPCLUST to represent the relationships among the samples based upon the expression data matrix of DEGs, DELs and DEMs, respectively.

### GO and KEGG pathway enrichment analysis of DEGs

GO enrichment analysis of DEGs was performed to identify the potential functional genes according to the GO database, which can organize genes into hierarchical categories and uncover the gene regulatory network on the basis of biological processes and molecular function [26]. KEGG pathway analysis of DEGs was carried out to ascertain the potential functions of these genes that participated in the pathways based on the KEGG database [27–29]. Fisher’s exact test was used to classify the GO category and identify the significant genes through the *P*-value and FDR [30].

### Target genes and lncRNAs prediction of DEMs

Three algorithms, miRanda (http://www.microrna.org/), Targetscan (http://www.targetscan.org/) and miRWalk (http://129.206.7.150/) were used for miRNA-mRNA target gene prediction. The target genes that overlapped with genes that were identified by GO and KEGG pathway enrichment analyses were considered as the predicted results. MiRanda and PITA (https://genie.weizmann.ac.il/pubs/mir07/mir07_exe.html) were used to predict target lncRNAs, and those that overlapped with DELs between sarcomas and normal muscle samples were used for the construction of the ceRNA network.

### Construction of the ceRNA network

**Fig. 1:**
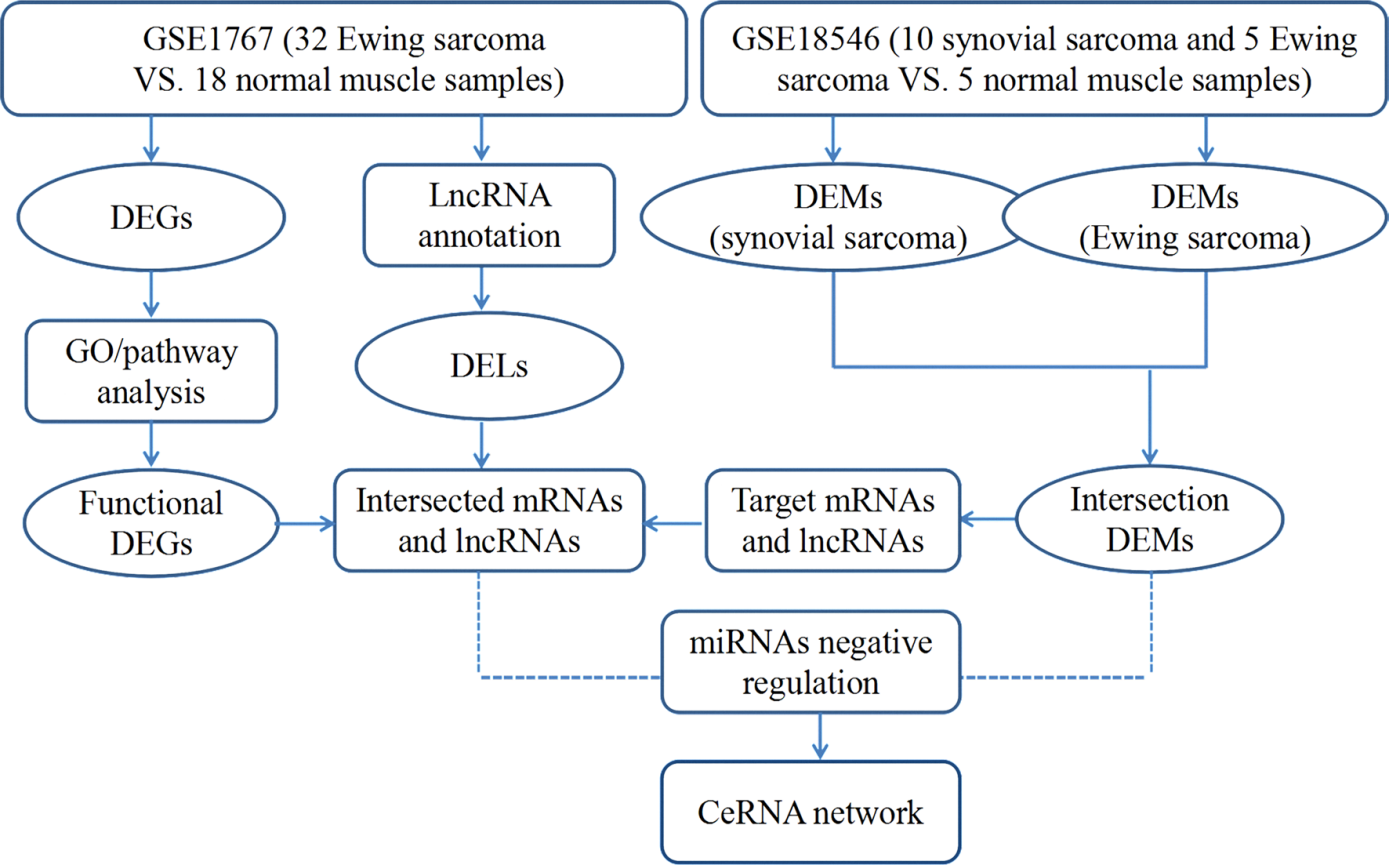
Flow chart of constructing the ceRNA network

Based on the ceRNA theory that lncRNA can served as an endogenous “sponge” to regulate the expression of mRNA by sinking miRNA [31], upregulated or downregulated miRNAs, and lncRNAs or mRNAs that are inverse relationships with miRNAs in the miRNA-mRNA and lncRNA-miRNA interaction pairs were chose to construct the lncRNA–miRNA–mRNA ceRNA network [32]. The ceRNA network was constructed and visualized using Cytoscape software (version 2.8.2) [33]. The flow chart, shown in Fig. 1, represents the overall process of ceRNA network construction.

### PPI network analysis

The PPI network of the DEGs that were included in the ceRNA network was constituted by using the STRING database version 11.0 (https://string-db.org/). Cytoscape software (version 2.8.2) was used to visualize the PPI network with a combined score of protein pairs > 0.4 as the cut-off value [34].

### Survival analysis

High-throughput experimental data with survival profiles of sarcoma patients was extracted from the TCGA database. The associations between biomarkers in the ceRNA network and overall survival of sarcoma patients were evaluated by using log rank test, with *P*-values less than 0.05 recognized as statistically significant.

## Results

### Available datasets of sarcomas

There were four datasets (GSE55625, GSE31045, GSE17674 and GSE18546) obtained from GEO, whereas the GSE55625 and GSE31045 datasets, with multiple zero and negative expression values, were excluded. The GSE17674 dataset based on the array platforms of Affymetrix Human Genome U133 Plus 2.0 Array contains 32 Ewing sarcoma and 18 normal muscle samples, from which we extracted probes for lncRNA annotation. The GSE18546 dataset includes 10 synovial sarcoma, 5 Ewing sarcoma and 5 normal muscle samples.

### Identification of DEGs, DELs and DEMs in sarcomas

DEGs and DELs were identified in 32 Ewing sarcoma samples compared with 18 normal muscle samples in the GSE17674 dataset. DEMs were identified in 10 synovial sarcoma and 5 Ewing sarcoma samples compared with normal muscle samples in the GSE18546 dataset. A total of 3415 mRNAs (2554 upregulated and 861 downregulated genes) and 338 lncRNAs (234 upregulated and 104 downregulated lncRNAs) were deferentially expressed in sarcoma patients of the GSE17674 dataset (Additional file 2: Table S1; Additional file 3: Table S2).

**Fig. 2:**
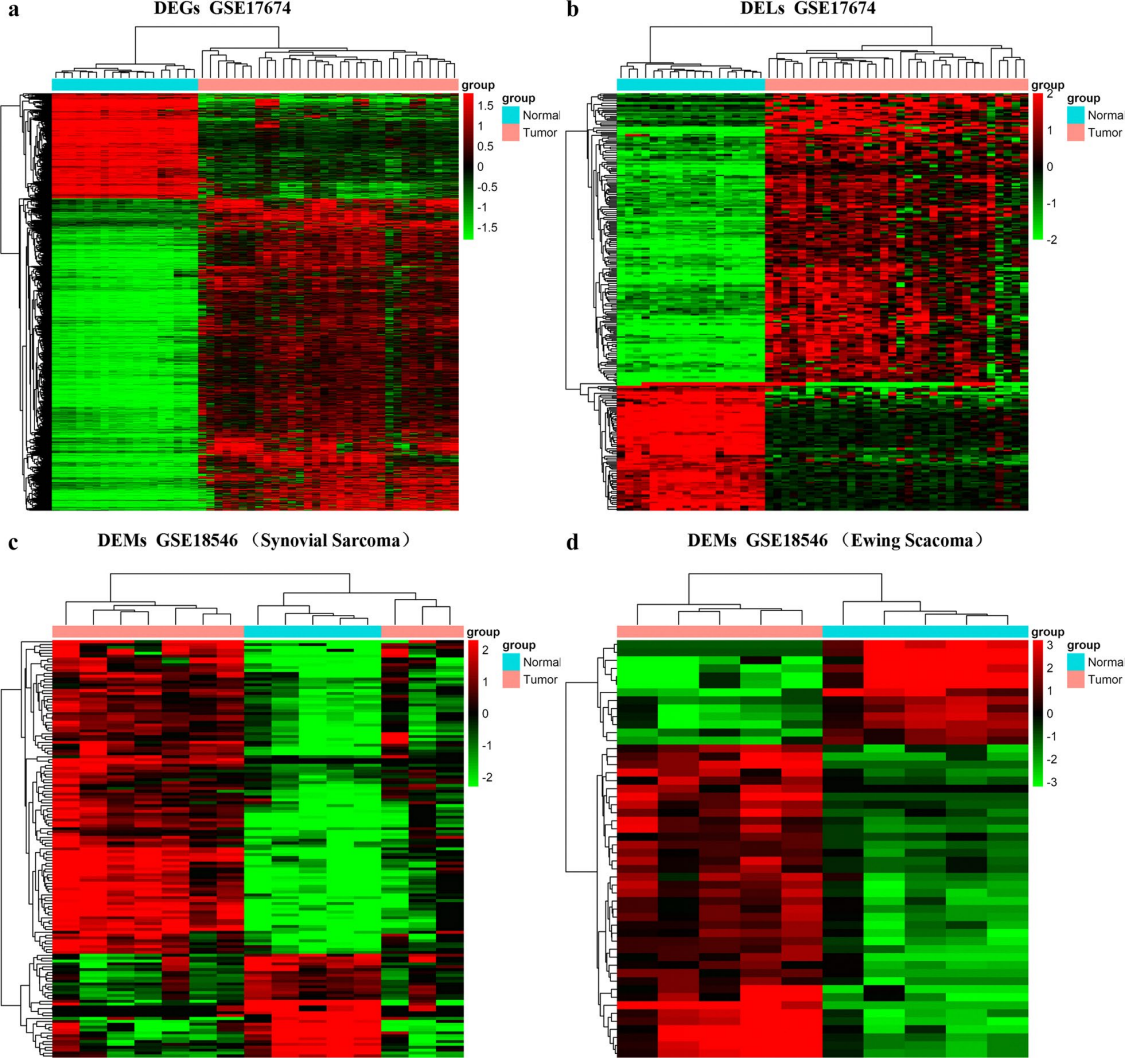
**a** The heatmap of DEGs between sarcoma and normal muscle samples. **b** The heatmap of DELs between sarcoma and normal muscle samples. **c** The heatmap of DEMs between synovial sarcoma and normal muscle samples. **d** The heatmap of DEMs between Ewing sarcoma and normal muscle samples. Blue represents a low expression level and red represents a high level

There were two sarcoma subtypes, Ewing sarcoma and synovial sarcoma, in the GSE18546 dataset, and two different expression miRNAs sets were identified. Fifty-two miRNAs (39 upregulated and 13 downregulated miRNAs) were differentially expressed in Ewing sarcoma and normal muscle samples. In addition, there were 145 (109 upregulated and 36 downregulated miRNAs) DEMs between synovial sarcoma and normal muscle samples. We created a Venn diagram intersecting the two DEM sets, and 26 upregulated DEM s and 10 downregulated DEMs were identified (Additional file 1: Fig. S1; Additional file 4: Table S3). Hierarchical clustering of the identified DEGs, DELs and DEMs was displayed as a heatmap (Fig. 2).

### GO and KEGG enrichment analysis of DEGs

**Fig. 3:**
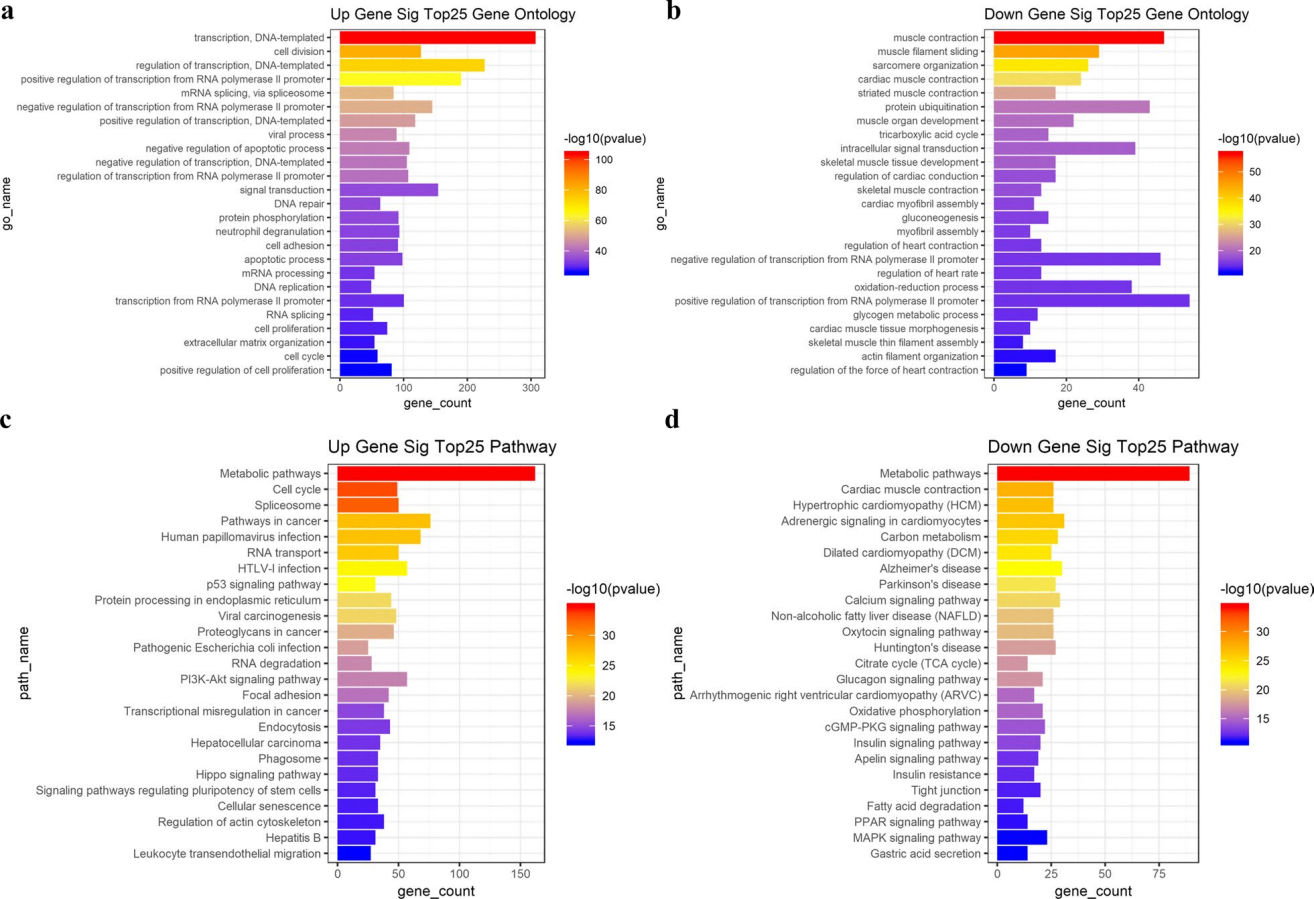
Top 25 enriched DEGs from the GO and KEGG pathway analyses. The bar plot shows the enrichment scores of GO and KEGG pathways. Different colors of bars represent different P values

GO enrichment analysis and KEGG pathways analysis of 3415 DEGs were performed to identify the potential functional genes. The upregulated and down-regulated genes were analyzed, respectively. The top 25 significantly enriched upregulated DEGs are presented in Fig. 3a, including transcription, DNA-templated (GO:0006351; *P* = 2.64 × 10^–104^), cell division (GO:0051301; *P* = 2.57 × 10^–82^) and regulation of transcription, DNA-templated (GO:0006355; *P* = 7.99 × 10–75). The most significantly enriched downregulated DEGs are presented in Fig. 3b, including muscle contraction (GO:0006936; *P* = 1.89 × 10^–57^), muscle filament sliding (GO:0030049; *P* = 5.78 × 10^–46^) and sarcomere organization (GO:0045214; *P* = 8.40 × 10^–38^). The main pathways for both the upregulated genes and the downregulated genes are metabolic pathways (Fig. 3c, d). The significant GO terms and pathways of upregulated and downregulated DEGs are presented in Additional file 5: Table S4 and Additional file 6: Table S5, respectively.

Finally, 1296 intersecting DEGs were extracted from the significantly enriched genes in GO and KEGG pathway analyses, which involved the upregulated and downregulated genes (Additional file 7: Table S6).

### Target genes and lncRNAs of DEMs

In this study, we have identified 36 DEMs, and we focused on whether these miRNAs would target the 1296 DEGs and 338 DELs. Based on the predicted targets of DEMs, 448 miRNA-mRNA interactor pairs (including 34 miRNAs and 269 mRNAs) and 454 lncRNA-miRNA interaction pairs (including 36 miRNAs and 117 lncRNAs) were obtained.

### ceRNA network

**Fig. 4:**
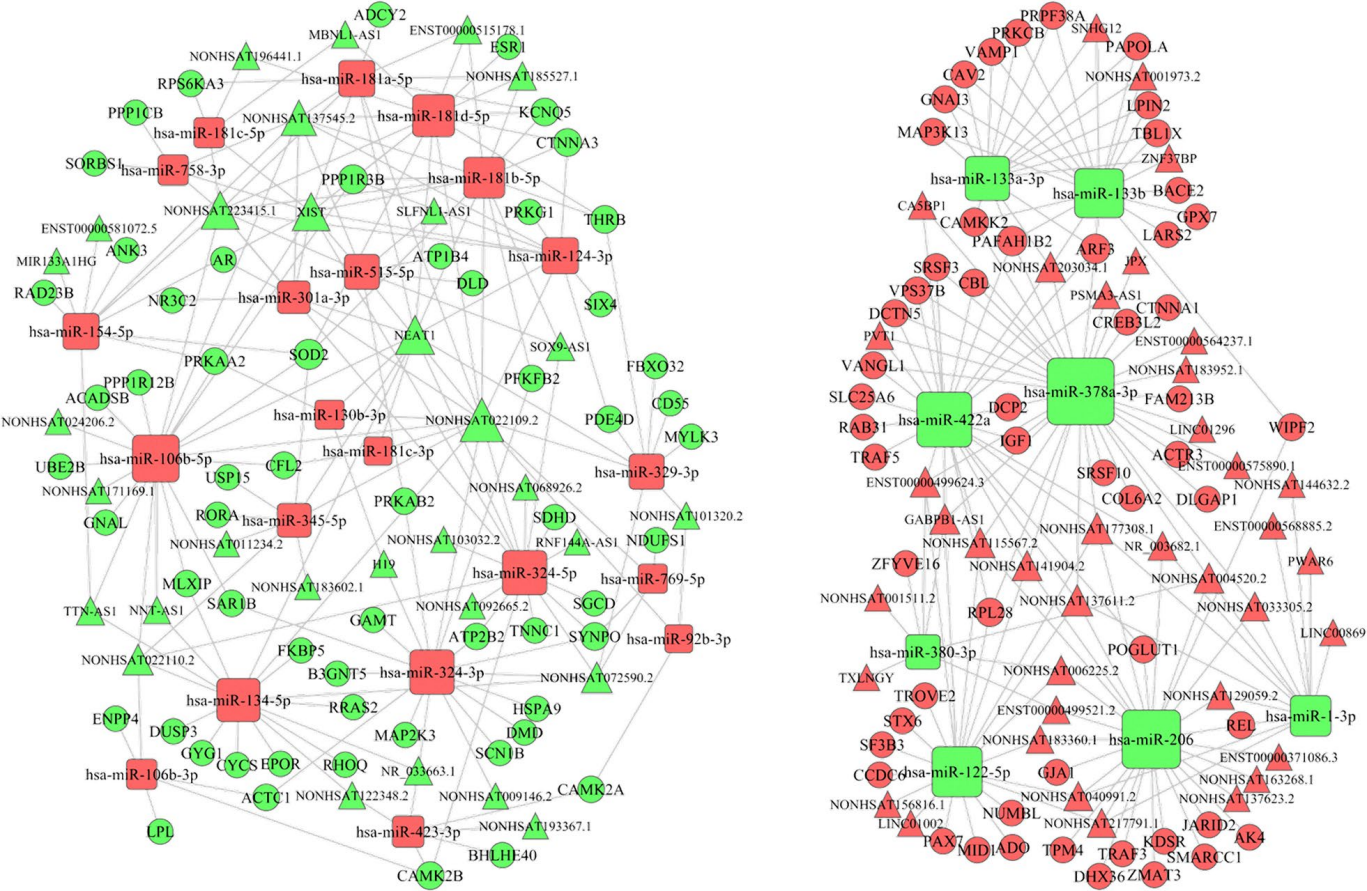
The lncRNA–miRNA–mRNA ceRNA network. Rectangles, circles and triangles represent miRNAs, mRNAs and lncRNAs, respectively. Red and green color represent upregulated and downregulated biomarkers in sarcomas. Gray lines indicate interactions between miRNA-mRNA and miRNA-lncRNA

According the ceRNA theory, we used the shared miRNA as a junction, that is, upregulated miRNAs, accompany with downregulated lncRNAs and mRNAs, and downregulated miRNAs, accompany with upregulated lncRNAs and mRNAs in the miRNA-mRNA and miRNA-lncRNA interaction pairs, to constructed a lncRNA–miRNA–mRNA ceRNA network. Finally, the ceRNA network was constructed with 1440 interactions, including 29 miRNAs, 69 lncRNAs and 113 mRNAs (Fig. 4; Additional file 8: Table S7).

### PPI network analysis

**Fig. 5:**
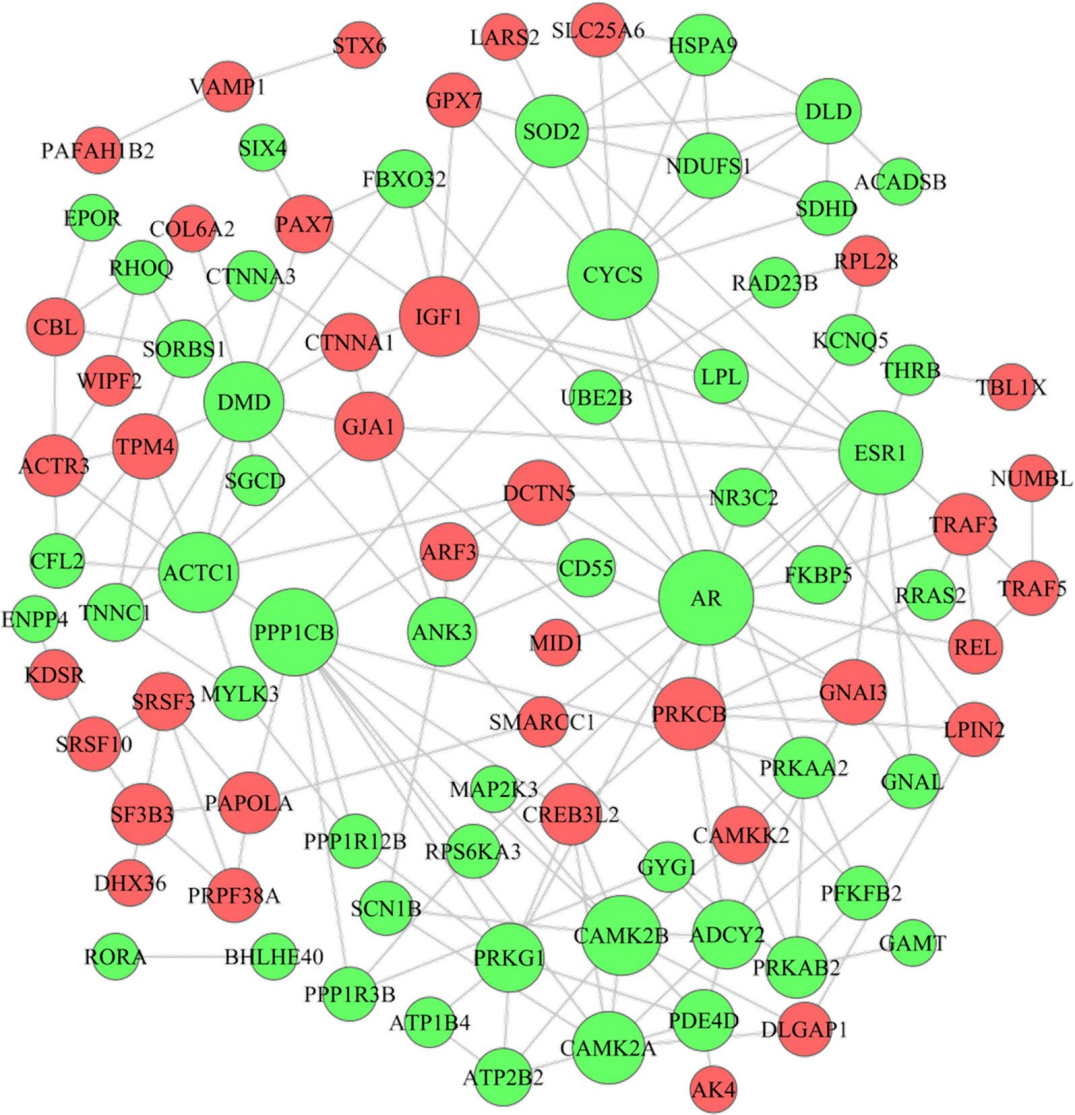
PPI network of DEGs created by STRING. Red and green nodes represent upregulated and downregulated proteins, respectively. The edge between nodes represents the interaction between two connected proteins

To further explore the most significant clusters of DEGs in the ceRNA network, we conducted PPI network analysis using the STRING database version 11.0 and visualization by Cytoscape. The most significant hub upregulated proteins were IGF1, PRKCB and GNAI3, and the most significant hub downregulated proteins were AR, CYCS and PPP1CB in the PPI network (Fig. 5; Additional file 9: Table S8).

### Survival analysis

**Fig. 6:**
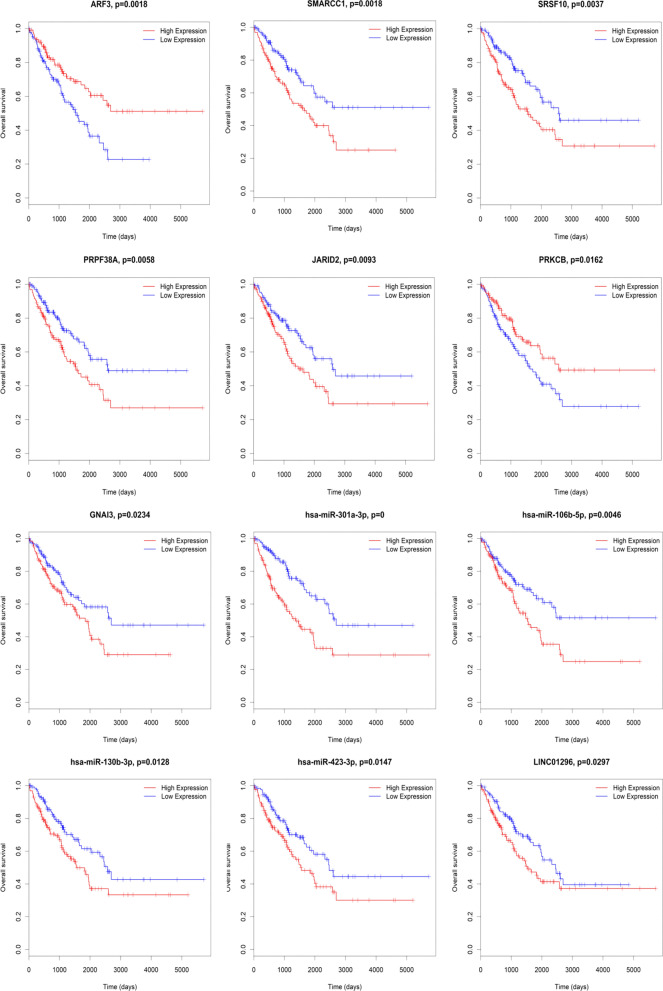
Kaplan–Meier survival curves of lncRNAs, miRNAs and mRNAs associated with overall survival of sarcomas patients

Furthermore, we performed the survival analysis based on the lncRNAs, miRNAs and mRNAs that were involved in the ceRNA network. The results show that seven mRNAs, four miRNAs and one lncRNA were significantly associated with the overall survival of sarcoma patients (*P* < 0.05). Among them, high expression levels of SMARCC1, SRSF10, PRPF38A, JARID2, GNAI3, miR-301a-3p, miR-106b-5p, miRNA-130b-3p, miR-423-3p and LINC01296 and low expression levels of ARF3 and PRKCB were associated with shorter overall survival in sarcomas (Fig. 6).

## Discussion

Increasing evidence shows that lncRNAs and miRNAs were differentially expressed and implicated in series of molecular processes, including differentiation, proliferation, metastasis, and transcriptional regulation, in sarcomas [35, 36]. However, the entire regulatory network that links the functions of coding and noncoding RNAs has not been extensively discussed. In the present study, bioinformatics analysis was utilized to integrate available sequencing datasets of sarcomas, and 1296 DEGs were identified in sarcoma samples by combining the GO and KEGG pathway enrichment analyses. A total of 338 DELs were discovered after the probes were reannotated, and 36 DEMs were ascertained through intersecting two different expression miRNAs sets. Further, 448 miRNA–mRNA interactions and 454 lncRNA–miRNA interactions were obtained through target gene prediction, and then, we constructed a lncRNA–miRNA–mRNA ceRNA network that contained 29 miRNAs, 69 lncRNAs and 113 mRNAs.

The ceRNA network identified in our study provided useful clues for further study. According to the DEGs in the ceRNA network, we constructed a PPI network, which showed the upregulated hub nodes, including IGF1, PRKCB and GNAI3, and the downregulated hub nodes, including AR, CYCS and PPP1CB. In addition, there were twelve RNAs in the ceRNA network associated with the prognosis of sarcomas based on the TCGA database. Among the seven survival-associated mRNAs, the high expression levels of SMARCC1, SRSF10, PRPF38A, JARID2 and GNAI3 were significantly associated with shorter overall survival in sarcomas (*P* = 0.0018, *P* = 0.037, *P* = 0.0058, *P* = 0.0093, and *P* = 0.0234, respectively). However, the high expressions of ARF3 and PRKCB were significantly associated with longer overall survival (*P* = 0.0018 and *P* = 0.0162), indicating that ARF3 and PRKCB overexpression could be positive prognostic factors in sarcoma patients. In these overall survival-associated mRNAs, GNAI3 and PRKCB, with the highest degree of connectivity, were hubs and tended to be essential [37]. Moreover, the PPI network showed that GNAI3 and PRKCB interacted with each other. The protein encoded by PRKCB is one of the PKC family members that has been reported to be involved in many different cellular functions. Surdez et al.’s study showed PRKCB is strongly overexpressed in Ewing sarcoma. PRKCB inhibition significantly increased apoptosis in Ewing sarcoma cells and prevented tumor growth in vivo [38]. GNAI3 encodes an alpha subunit of guanine nucleotide-binding proteins and is involved in various transmembrane signaling pathways. In addition, in the ceRNA network, both GNAI3 and ARF3 can be regulated by miR-133b and miR-133a-3p, which are at the center of the regulatory network and interact with lncRNAs including NONHSAT001973.2, NONHSAT203034.1, SNHG12 and ZNF37BP. Recent research has demonstrated that SNHG12 was significantly overexpressed in osteosarcoma, and high expression of SNHG12 tended to lead to a poor prognosis of osteosarcoma patients. Researchers further confirmed that SNHG12 promoted tumorigenesis and metastasis by activating the Notch signaling pathway, wherein it functioned as a ceRNA, modulating the expression of Notch2 by competing with miR-195-5p [39]. Therefore, the interaction of SNHG12, miR-133a-3p/miR-133b and GNAI3 in sarcoma was worthwhile of exploration. Additionally, ARF3 could also be regulated by miR-378a-3p and miR-422a, and among them, miR-378a-3p targeted LINC01296. It has been revealed that the high expressions were significantly associated with shorter overall survival of sarcoma patients (*P* = 0.0297) through our survival analysis.

The high expressions of miRNA-301a-3p, miRNA-106b-5p, miRNA-130b-3p, and miRNA-423-3p were significantly associated with a shorter overall survival of sarcoma patients (*P* < 0.0001, *P* = 0.0046, *P* = 0.0128, and *P* = 0.0147, respectively). The overexpression of miR-301a was revealed in Ewing sarcoma cell lines. Additionally, the transfection of anti-miR-301a inhibited the proliferation and cell cycle progression of Ewing sarcoma cells [40]. In the ceRNA network, the NEAT1/miR-301a-3p, XIST/miR-301a-3p, miR-301a-3p/NR3C2, and miR-301a-3p/AR interactions were identified. XIST was significantly upregulated in osteosarcoma tissues and cell lines, and increased XIST expression was associated with the poor overall survival of patients [41]. Nakatani et al. have defined miR-130b as an independent predictor of risk for disease progression and survival by microarray analysis [42]. Until now, there is no study that reports the association of miRNA-423-3p and miRNA-106b-5p with sarcomas. The NEAT1/miR-301a-3p/AR, XIST/miR-301a-3p/AR, NEAT1/miR-301a-3p/NR3C2, and XIST/miR-301a-3p/AR axes predicted by the construction of the ceRNA network may provide a more precise research direction for exploring biological mechanisms extending from this ceRNA network.

There were several limitations in the present study. Sarcomas contain multiple distinct subtypes, and only two subtypes were involved in our study. More RNA sequencing datasets of all subtypes of sarcomas are required for the construction of a ceRNA network of sarcomas or a particular subtype. Furthermore, regulatory pathways of the ceRNA network are very complex, and ceRNA activity is influenced by multiple factors, such as the abundance and subcellular location of ceRNA components, miRNA/ceRNA affinity, RNA editing and RBPs [9]. This study only investigated the putative interactions of lncRNAs, miRNAs and genes, which requires further validation.

## Conclusion

In conclusion, our work successfully constructed a ceRNA network by bioinformatics analysis based on the GEO database, providing a comprehensive resource for investigating the ceRNA regulation in sarcomas. Importantly, we screened out candidate prognostic biomarkers that are involved in the ceRNA network, which may exhibit important roles in the therapeutic target and prognosis analysis in sarcoma patients.

## Supplementary Information

**Additional file 1. Fig. S1**: Common DEMs of Ewing sarcoma and synovial sarcoma comparisons in the expression profile of GSE18546.

**Additional file 2. Table S1**: DEGs between sarcoma samples and normal muscle samples.

**Additional file 3. Table S2**: DELs between sarcoma and normal muscle samples.

**Additional file 4. Table S3**: The intersection of significantly DEMs of Ewing sarcoma and synovial sarcoma comparisons.

**Additional file 5. Table S4**: Significantly enriched GO terms of DEGs.

**Additional file 6. Table S5**: Significantly enriched pathway terms of DEGs.

**Additional file 7. Table S6**: The intersecting DEGs of GO and KEGG pathway enrichment.

**Additional file 8. Table S7**: The lncRNA–miRNA–mRNA ceRNA network of sarcomas.

**Additional file 9. Table S8**: The PPI nodes of DEGs involved in the ceRNA network.

## Data Availability

The dataset supporting this article’s conclusions is available in the NCBI GEO repository, with accession number GSE17674 in https://www.ncbi.nlm.nih.gov/geo/query/acc.cgi?acc=GSE17674 and GSE18546 in https://www.ncbi.nlm.nih.gov/geo/query/acc.cgi?acc=GSE18546. The survival data of sarcomas patients were obtained from https://gtexportal.org/home/datasets.

## Abbreviations

GEO: Gene expression omnibus
DEGs: Differentially expressed genes
DELs: Differentially expressed lncRNAs
DEMs: Differentially expressed miRNAs
GO: Gene ontology
KEGG: Kyoto encyclopedia of genes and genomes
PPI: Protein–protein interaction
FDR: False discovery rate
TCGA: The cancer genome atlas
