# Supplementary figures and images for: Integrated analysis of lncRNA–miRNA–mRNA ceRNA network and the potential prognosis indicators in sarcomas
Source: BMC Med Genomics. 2021 Mar 2;14:67. doi: 10.1186/s12920-021-00918-x (PMC7927383; doi:10.1186/s12920-021-00918-x)

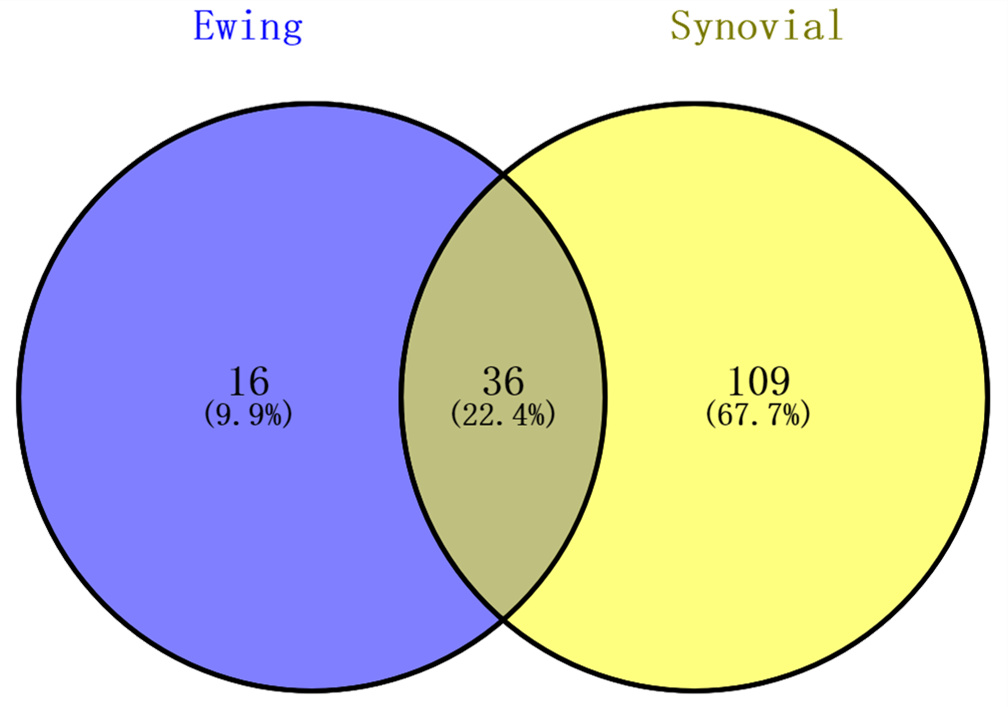

Supplement: Supplementary file 1 — Additional file 1. Fig. S1: Common DEMs of Ewing sarcoma and synovial sarcoma comparisons in the expression profile of GSE18546. [file 12920_2021_918_MOESM1_ESM.tif]
